# The HPV16 E7 Oncoprotein Disrupts Dendritic Cell Function and Induces the Systemic Expansion of CD11b^+^Gr1^+^ Cells in a Transgenic Mouse Model

**DOI:** 10.1155/2016/8091353

**Published:** 2016-07-11

**Authors:** Gabriela Damian-Morales, Nicolás Serafín-Higuera, Mario Adán Moreno-Eutimio, Enoc M. Cortés-Malagón, José Bonilla-Delgado, Genaro Rodríguez-Uribe, Rodolfo Ocadiz-Delgado, Paul F. Lambert, Patricio Gariglio

**Affiliations:** ^1^Department of Genetics and Molecular Biology, Centro de Investigación y de Estudios Avanzados del Instituto Politécnico Nacional (CINVESTAV-IPN), 07360 Mexico City, CDMX, Mexico; ^2^Unit of Health Sciences, Faculty of Odontology, Universidad Autónoma de Baja California, 21100 Mexicali, BC, Mexico; ^3^Research Unit, Hospital Juárez de México, 07760 Mexico City, CDMX, Mexico; ^4^McArdle Laboratory for Cancer Research, University of Wisconsin, School of Medicine and Public Health, Madison, WI 53726, USA

## Abstract

*Objective.* The aim of this study was to analyze the effects of the HPV16 E7 oncoprotein on dendritic cells (DCs) and CD11b^+^Gr1^+^ cells using the K14E7 transgenic mouse model.* Materials and Methods.* The morphology of DCs was analyzed in male mouse skin on epidermal sheets using immunofluorescence and confocal microscopy. Flow cytometry was used to determine the percentages of DCs and CD11b^+^Gr1^+^ cells in different tissues and to evaluate the migration of DCs.* Results.* In the K14E7 mouse model, the morphology of Langerhans cells and the migratory activity of dendritic cells were abnormal. An increase in CD11b^+^Gr1^+^ cells was observed in the blood and skin of K14E7 mice, and molecules related to CD11b^+^Gr1^+^ chemoattraction (MCP1 and S100A9) were upregulated.* Conclusions.* These data suggest that the HPV16 E7 oncoprotein impairs the function and morphology of DCs and induces the systemic accumulation of CD11b^+^Gr1^+^ cells.

## 1. Introduction

High-risk human papillomaviruses (HR-HPVs) infect squamous epithelia and promote proliferative lesions. Moreover, persistent HR-HPV infection can eventually lead to the development of epithelial cancers (i.e., cervical, vulvar, oropharyngeal, anal, and penile) [1]. The E6 and E7 oncoproteins are the main regulators of the carcinogenic potential of HR-HPVs [2], which modulate various components of the immune system [3]. To gain insight into the role of the HPV16 E7 protein in the regulation of hyperplasia-associated inflammation, we utilized a previously well-characterized transgenic mouse model expressing the HPV16 E7 oncoprotein (K14E7 mice) [4]. Interestingly, it has been reported that the hyperplastic skin of the K14E7 mouse is prone to premalignant skin lesions caused in part by a local immunosuppressive environment [5–7]. This phenotype is characterized by altered cytokine and chemokine expression [8, 9], which results in the recruitment of immune cells [8, 10].

Dendritic cells (DCs) are antigen-presenting cells that control the host immune response. DCs localize to different tissues, and once they have captured pathogens, they migrate to the lymph nodes where they present antigens to lymphocytes [11]. CD11c is commonly used as a general marker of DCs in mice, as are the costimulatory molecules CD80 and CD86. Langerhans cells (LCs), a subset of DCs that localize to the epidermis, express markers such as MHC-II, CD205, and CD207 [12–14]. Importantly, HPV oncoproteins can affect the differentiation and density of DCs [6, 15, 16] and can disrupt the expression of DC markers.

Myeloid-derived suppressor cells (MDSCs) represent a heterogeneous population of immature myeloid cells that exhibit immunosuppressive activity. In mice, the molecular phenotype of these cells is CD11b^+^Gr1^+^ [17], and the infiltration and systemic expansion of these cells in chronic inflammation and tumors have been reported [18]. Additionally, an increased proportion of CD11b^+^Gr1^+^ cells was observed in immunodeficient mice receiving cervical cancer cell line-derived grafts [19].

The aim of this work was to assess the effects of the HPV16 E7 oncoprotein on two cell populations implicated in immune regulation, LCs and CD11b^+^Gr1^+^ cells, using the K14E7 mouse model. Our results demonstrate that the morphology of CD207^+^ cells in the mouse skin was disrupted and that migration activity was impaired. Additionally, increased proportions of CD11b^+^Gr1^+^ cells were observed in the skin and blood of K14E7 mice. Thus, it is possible that the effects of the HPV16 E7 oncoprotein can spread from local to distant sites and may promote an immunosuppressive environment in K14E7 mice.

## 2. Materials and Methods

### 2.1. Animals

The K14E7 and nontransgenic (NT) FvB/N mice have been described previously [4]. The K14E7 mice were bred and maintained on the FvB/N background. Male NT and K14E7 mice between the ages of 5 and 7 months were sacrificed by cervical dislocation. The animals were housed according to the international regulations of the Association for Assessment and Accreditation of Laboratory Animal Care (AAALAC). All mouse procedures were performed according to a protocol approved by the Research Unit for Laboratory Animal Care Committee (UPEAL-CINVESTAV-IPN, Mexico; NOM-062-ZOO-1999).

### 2.2. Epidermal Sheets

Epidermal sheets were obtained as previously described [20]. Briefly, shaved dorsal and ventral skin samples from K14E7 and NT mice were resected and incubated, dermis-side down, in 1x PBS/0.5 M EDTA at 37°C for 2 hours. The epidermis was then mechanically separated from the dermis and washed in 1x PBS. The resulting epidermal sheets were fixed in acetone for 20 minutes and then washed again. Epidermal sheets were used for hematoxylin and eosin staining or for immunohistochemistry (IHC).

### 2.3. Immunofluorescence (IF) and Immunohistochemistry (IHC)

For the IF procedure, epidermal sheets were rinsed in 1x PBS that was supplemented with 0.3% Triton X-100 and blocked for 2 hours at 4°C with background blocker (Diagnostic Biosystems, CA, USA); the epidermal sheets were then washed three times with 1x PBS and incubated overnight at 4°C with an anti-CD207 antibody (donated by Juliana Idoyaga, Ph.D., Rockefeller University). Next, the sheets were incubated with a Texas Red-labeled secondary antibody (Vector, Burlingame, CA, USA) for 2 hours at room temperature. Finally, the sheets were rinsed as described above, counterstained with DAPI, and mounted in Vectashield (Vector, Burlingame, CA, USA).

For IHC, the epidermal sheets were incubated with PolyDetector Peroxidase Block quenching buffer (Bio SB, CA, USA) and 0.1% BSA and then incubated with a primary antibody against MHC-II (1 : 50) (M5/114.15.2; eBioscience, CA, USA) or an anti-CD205 antibody (1 : 25) (NLDC-145; AbD Serotec, NC, USA). After the sheets were washed in 1x PBS, they were incubated with a secondary antibody (1 : 200) (anti-rat HRP; eBioscience, CA, USA). Proteins were detected using the DAB chromogen (0.3 mg/mL DAB; Bio SB, CA, USA). Sheets that had not been incubated with primary antibody were included as negative controls.

### 2.4. Digital Image Capture

The IF and IHC preparations were examined by confocal microscopy using SP2 microscope (Leica Microsystems, Wetzlar, Germany) and a DFC290 HD digital camera (Leica Microsystems, IL, USA), respectively. Captured images were imported into the ImageJ software program (version 1.37v, National Institutes of Health, Bethesda, MD) to produce maximum projections, and Adobe Photoshop (Adobe Systems, CA, USA) was used to equalize the brightness and contrast in all of the images.

### 2.5. Cell Suspensions

The spleens and lymph nodes (inguinal, popliteal, axillary, and brachial) from NT and K14E7 mice were resected. Single-cell suspensions were obtained after mechanical disaggregation using the Medimachine system (BD Biosciences, CA, USA), according to the manufacturer's instructions. Erythrocytes from blood (collected by cardiac puncture) and spleen samples were lysed using an ACK (ammonium-chloride-potassium) buffer. Skin samples were incubated in 1 mg/mL Dispase II (Sigma-Aldrich, MO, USA) and 0.5 M EDTA for 1 hour at 37°C, and then mechanical disaggregation was performed. All samples were filtered through a 70 *μ*m cell strainer and pelleted by centrifugation (600 ×g for 5 minutes at 4°C). Finally, the cells were suspended and analyzed using flow cytometry.

### 2.6. FITC Painting

The abdomens of shaved mice were painted with 200 *μ*L 0.5% FITC solution (Sigma-Aldrich, MO, USA) in a 1 : 1 ratio of acetone : dibutyl phthalate (Sigma-Aldrich, MO, USA). Twenty-four hours later, the inguinal, popliteal, axillary, and brachial lymph nodes were harvested. Single-cell suspensions were prepared as described above, and the cells were stained with anti-CD11c (APC, BD Biosciences, CA, USA) for flow cytometric analysis.

### 2.7. Flow Cytometry

Cell suspensions were counted, and 1 × 10^6^ cells were resuspended in 1x PBS containing 0.5% BSA. Staining was performed by incubating the cell suspensions with the appropriate antibodies for 20 minutes at 4°C. The following antibodies against mouse antigens were used: anti-CD11c-BV421 (BioLegend, CA, USA), anti-CD207-FITC (eBioRMUL.2, eBiosciences, CA, USA), anti-MHCII-FITC (BD Pharmingen, CA, USA), anti-CD11b-APC (CALTAG Laboratories, Buckingham, UK), and anti-Gr1-FITC (RB6-8Cs, eBiosciences, CA, USA). A minimum of 100,000 events were collected by flow cytometry (FACS Accuri C6, BD, Biosciences, CA, USA) and analyzed using the FlowJo software program V10.0.8 (Tree Star, CA, USA).

### 2.8. Western Blotting

Samples of murine skin were lysed in lysis buffer containing 1% Triton X-100, 1 M Tris-HCl (pH 7.4), 0.5 M EDTA, 2 M NaCl, 10% Na-deoxycholate, 10% sodium dodecyl sulfate (SDS), and glycerol for 30 minutes at room temperature. Proteins were separated by size on 15% polyacrylamide SDS gels (PAGE) and transferred to nitrocellulose membranes (BioRad, CA, USA) by electroblotting. After the membranes were blocked with 1x Animal-Free Blocker (Vector Laboratories, Inc., Burlingame, CA, USA) for 1 hour, they were incubated overnight with a primary antibody against S100A9 (1 : 5000) (Abcam, Cambridge, UK) and subsequently incubated with an anti-rat HRP-conjugated secondary antibody (1 : 15000) (Sigma-Aldrich, MO, USA). Signals were developed and visualized using the Clarity Western ECL Substrate (BioRad, CA, USA). The intensity of the protein band was analyzed using the ImageJ software program (version 1.41). Equal loading and transfer of the proteins were assessed using a monoclonal antibody against beta-actin (1 : 1000) (Sigma-Aldrich, MO, USA).

### 2.9. Determination of Cytokine Concentration

The concentrations of interleukin-1 beta (IL1*β*), IL6, IL12p70, tumor necrosis factor (TNF), interferon-gamma (IFN*γ*), and monocyte chemoattractant protein-1 (MCP1) in the skin were analyzed using a cytometric bead array (CBA) Mouse Inflammation Kit (BD, Biosciences, CA, USA) according to the manufacturer's recommended protocol [21]. Briefly, shaved skin samples were dissected, weighed, homogenized by mechanical disaggregation with 1x PBS at 4°C, and centrifuged (600 ×g for 5 minutes at 4°C). The recovered supernatant was incubated with a mixture of capture beads and PE fluorochrome detection reagent for 2 hours at 4°C. After the beads were washed with 1x PBS, the concentration of cytokines was determined by measuring the unique fluorescence intensity of each bead using flow cytometry (FACS Accuri C6, BD, Biosciences, CA, USA) and the FCAP Array V3.0 software program (Soft Flow, Inc., USA).

### 2.10. Statistical Analysis

Statistical comparisons were carried out using unpaired Student's *t*-test. *p* values ≤ 0.05 were considered statistically significant.

## 3. Results

### 3.1. Hyperplastic Skin from Mice Expressing the HPV16 E7 Oncoprotein Contains LCs with Altered Morphology

**Figure 1:**
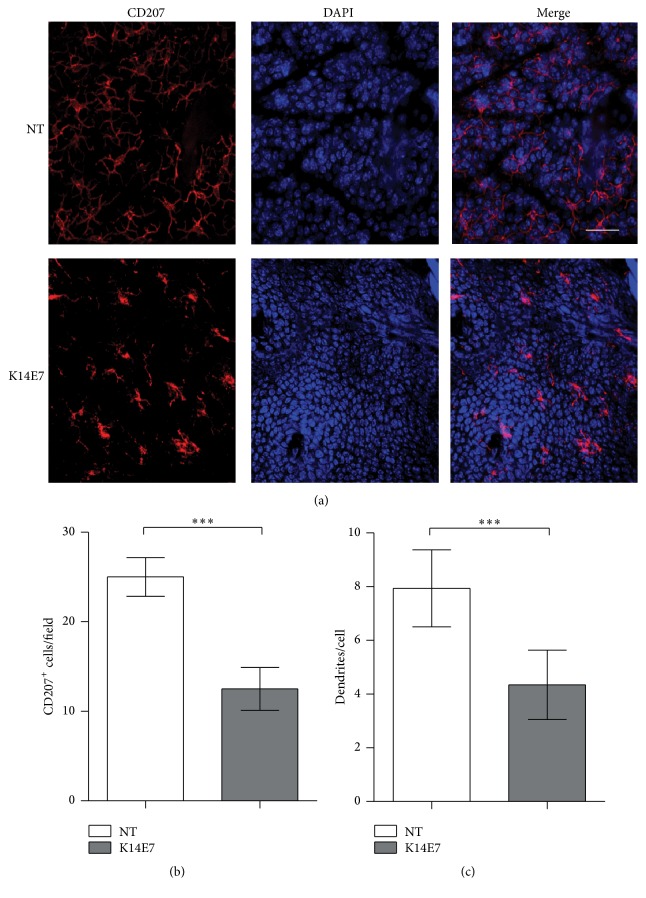
CD207^+^ cells detected in mouse epidermis. (a) Epidermal sheets of nontransgenic (NT) and K14E7 mice were stained with anti-CD207 antibody, and cell nuclei were counterstained with DAPI. Magnification, 63x. Scale bar = 30 *μ*m. (b) Numbers of CD207^+^ cells per field and (c) dendrites on CD207^+^ cells were counted using confocal microscopy. Significant differences are indicated with asterisks: ^*∗∗∗*^ *p* < 0.001.

To analyze the morphology of LCs, the CD207 marker was examined by IF. In epidermal sheets from K14E7 mice, LCs were more rounded in shape (Figure 1(a)), displayed fewer cells per field (Figure 1(b)), and were less dendritic in appearance (Figure 1(c)). Additionally, we detected CD205 and MHCII using IHC and obtained similar results (see Supplementary Figure in Supplementary Material available online at http://dx.doi.org/10.1155/2016/8091353).

### 3.2. Percentage of DCs in Different Tissues of K14E7 Transgenic Mice

**Figure 2:**
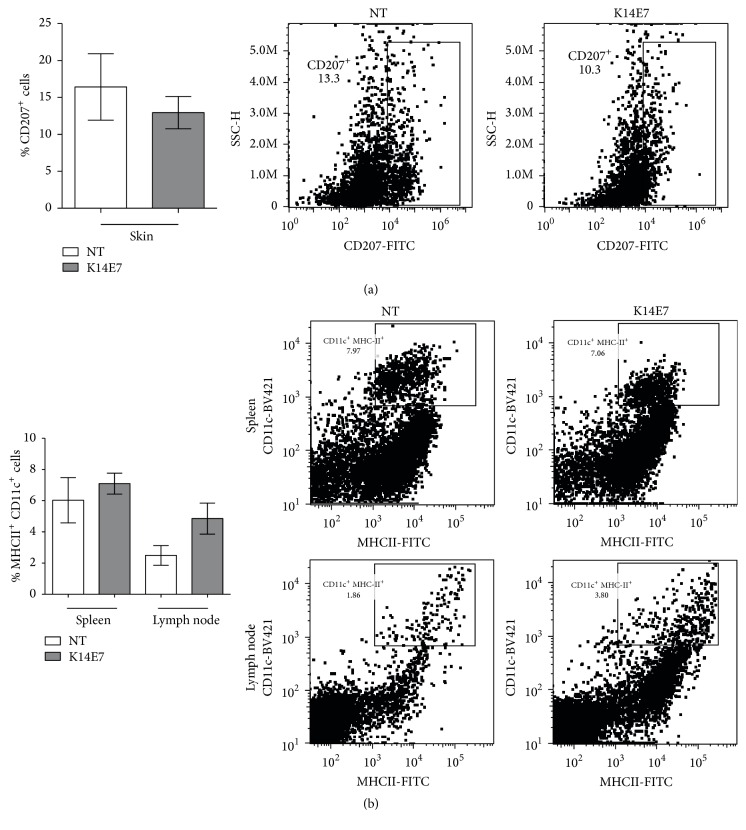
Dendritic cells from the skin, spleen, and lymph nodes of K14E7 mice. The percentage of CD207^+^ cells in the skin (a) and percentage of CD11c^+^MHCII^+^ cells in spleen and node lymphoid tissues (b) of transgenic mice (K14E7) and nontransgenic mice (NT). Representative dot plots of flow cytometry are shown. Data obtained from 12 mice are shown (*n* = 6 mice per experimental group). Experiments were performed in duplicate at least 2 times.

Although a reduced proportion of LCs (CD207^+^ cells) was observed in skin from transgenic mice relative to NT mice, this difference was not statistically significant (Figure 2(a)). The percentage of DCs in other tissues, including the spleen and lymph nodes, was also analyzed. No significant differences in the proportions of CD11c^+^MHCII^+^ DCs were detected.

### 3.3. Impaired Migration of DCs to the Lymph Nodes in K14E7 Mice

**Figure 3:**
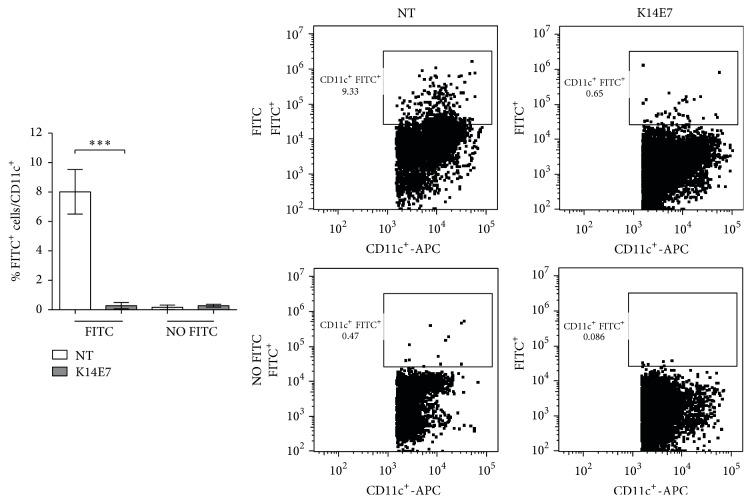
Impaired migration of dendritic cells from the skin to the lymph nodes in K14E7 mice after sensitization with FITC. Shaved ventral mouse skin from K14E7 and NT mice was painted with fluorescein isothiocyanate (FITC) or vehicle control alone (NO FITC). After 24 hours, the inguinal, popliteal, axillary, and brachial lymph nodes were obtained and disaggregated. FITC^+^/CD11c^+^ cells were analyzed using flow cytometry. Experiments were performed in duplicate at least 2 times (*n* = 4 mice per experimental group). Significant differences are indicated with asterisks: ^*∗∗∗*^ *p* < 0.001.

To evaluate migratory activity, DCs were analyzed in the lymph nodes of K14E7 and NT mice after painting the ventral skin with FITC-labeled solution. Interestingly, impaired cell migration was observed exclusively in mice expressing the E7 oncoprotein; a decreased percentage of CD11c^+^FITC^+^ cells was observed in the lymph nodes of these mice (Figure 3).

### 3.4. Increased Percentage of CD11b^+^Gr1^+^ Cells in K14E7 Transgenic Mice

**Figure 4:**
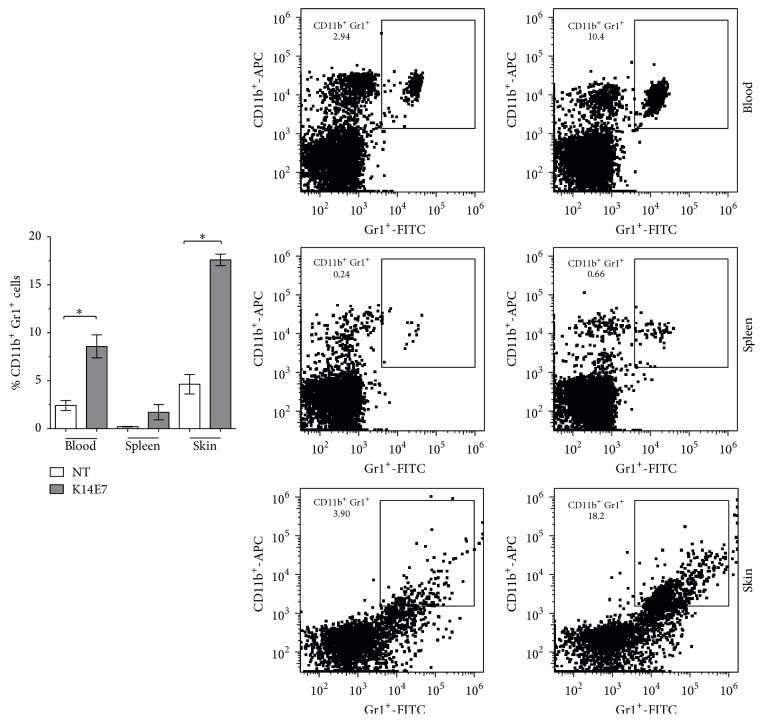
CD11b^+^Gr1^+^ cells in K14E7 mice. The proportion of CD11b^+^Gr1^+^ cells in the blood, spleen, and skin of nontransgenic (NT) and K14E7 mice was evaluated using flow cytometry. Representative flow cytometry data of 3 independent experiments are shown (*n* = 3 mice per group). Significant differences are indicated with an asterisk: ^*∗*^ *p* < 0.05.

**Figure 5:**
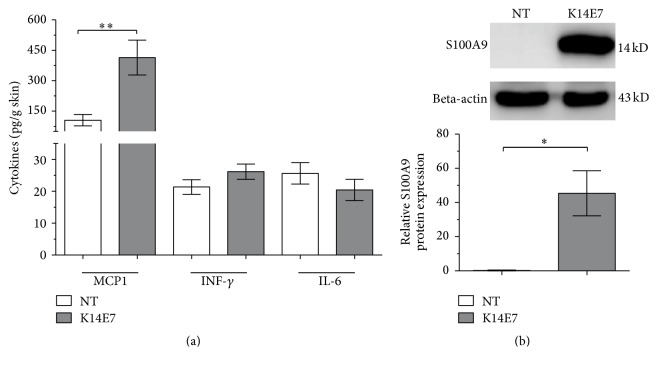
MCP1, INF-*γ*, IL-6, and S100A9 expression levels in K14E7 mouse skin. (a) MCP1, INF-*γ*, and IL-6 protein levels in skin cells from nontransgenic (NT) and K14E7 mice were evaluated by cytometric bead array using flow cytometry (*n* = 3 mice per experimental group). (b) Expression of S100A9 in NT and K14E7 mouse skin as detected by Western blotting; beta-actin expression was used as a loading control. Experiments were performed in duplicate at least 2 times. Significant differences are indicated with asterisks: ^*∗*^ *p* < 0.05, ^*∗∗*^ *p* < 0.01.

A systemic expansion of CD11b^+^Gr1^+^ cells has previously been reported in K14HPV16 mice [22]. To evaluate whether the E7 oncoprotein exerts a similar effect, CD11b^+^Gr1^+^ cells were investigated in the K14E7 mouse model. We observed an increase in the number of CD11b^+^Gr1^+^ cells in the skin and blood of K14E7 mice compared with NT mice (Figure 4). As the inflammatory profile is related to the increase in CD11b^+^Gr1^+^ cells, a cytokine panel was measured in skin cell homogenates. We only observed an increase in the expression levels of MCP1 (Figure 5(a)) in K14E7 mice compared with NT mice.

Given that CD11b^+^Gr1^+^ cells synthesize the S100A9 protein [23] and are attracted to MCP1 [24, 25], we also analyzed the levels of S100A9 by Western blot. Interestingly, S100A9 was overexpressed in the skin of K14E7 transgenic mice compared with NT mice (Figure 5(b)).

## 4. Discussion

Morphological alterations in LCs have been observed in patients infected with HR-HPV [26]. In addition, a recent study reported that CD207^+^ cells in the skin of K14E7 mice exhibit a round shape and a less dendritic appearance [14]. Our findings reinforce these previous reports. Although the precise causes of the morphological alterations we observed are unclear, Cdc42 (Rho-family GTPase) is known to be important for LC morphology given that Cdc42-deficient LCs from mouse skin appear as round cell bodies with few visible dendrites [27, 28]. Therefore, to explain the effect of the E7 oncoprotein on LC morphology, detailed molecular studies of Cdc42 in the K14E7 mouse model are required.

We also studied the effect of E7 oncoprotein on the percentage of LCs; however, we did not observe changes in the percentage of LCs in the skin of transgenic mice compared to controls.

In contrast to our results, Abd Warif et al. [14] showed that the percentage of LCs in the skin of K14E7 mice increases. We believe that these discrepancies are probably due to the genetic background used. Notably, Flacher et al. [29] reported a lower density of LCs on epidermal sheets in the C57BL/6 strain compared to the BALB/c strain. Furthermore, the number of DCs obtained from lymph nodules in the BALB/c strain was twofold greater than that in the C57BL/c strain. Furthermore, a subgroup of DCs is only present in BALB/c and 129/Sv strains [29].

Additionally, factors such as gender, age, or tumor susceptibility cannot be ignored in order to explain differences between our genetic background and the C57BL/6 strain. For example, K14E7 mice with a FvB/N background are more susceptible to tumor development in comparison to K14E7 mice with a FvB7/N-C57BL/6 double genetic background [30, 31].

Furthermore, it is important to mention that the apparent discrepancy between Figures 1(b) and 2(a) could be due to an increase in the epidermal thickness in K14E7 mice. This increased thickness may obscure the full visual field needed to observe all the CD207^+^ cells in the epidermal sheets. We would like to emphasize that the primary purpose for using confocal microscopy was to observe the morphology of the CD207^+^ cells. Strictly, this technique is not the best way to quantify cells; therefore, our best conclusion about the proportion of CD207^+^ cells was taken from our data obtained by flow cytometry.

The migration of DCs to lymph nodes is important for maintaining homeostasis in the skin; however, this balance is affected by several conditions, such as HPV persistence [7, 9]. Our results revealed that DC (CD11c^+^ FitC^+^) migration from skin to lymph nodes was impaired. Similar to our data, a reduction in LC migration in K14E7 mice was recently reported [14]. In addition, LCs from neonatal mouse skin—with morphologic alterations and reduced antigen uptake—demonstrated limited migration capabilities that contributed to antigen-specific tolerance [13].

We did not characterize the population of CD11c^−^ FITC^+^ cells that migrated into the lymph node in the experiments for Figure 3. However, the fact that other cells such as macrophages have the ability to migrate into the lymph nodes from the skin should be taken into account [32]. Therefore, additional studies should be performed to evaluate this alternative in K14E7 mice.

Finally, although CD11c and MHCII are widely used as the sole markers for DCs, we cannot rule out the possibility that our data for DCs include a fraction of CD11c^+^MHCII^+^F4/80^+^ cells (macrophages).

To date, accumulating evidence has suggested that a heterogeneous group of immature myeloid cells play an important role in promoting an immunosuppressant environment in several types of cancer; these cells are characterized by the immunophenotype of CD11b^+^Gr1^+^ cells in mice [17]. We also studied the proportion of CD11b^+^Gr1^+^ cells in different tissues of the K14E7 mouse model. Similar to our study, mice expressing the early region of HPV16 under the control of the K14 promoter (K14HPV16) exhibited an increased proportion of CD11b^+^Gr1^+^ cells in the skin [33]. This result could be explained by the upregulated expression of soluble factors, which possibly mediate the inflammatory environment. For example, increased production of IFN*γ*, IL18, IL17, and IL23 and decreased levels of IL6 and IL1*β* have been reported in the skin of K14E7 transgenic mice [7, 9]. Additionally, an increase in the expression of MCP1 and S100A9 in the ear skin and cervix of K14E7 (treated with estradiol) mice, respectively, was previously reported [8, 34]. It has been reported that both MCP1 and S100A9 mediate the migration and infiltration of CD11b^+^Gr1^+^ cells into inflammatory sites and tumor tissues [24, 25] and inhibit DC differentiation [35] and function [36]. Consistent with this finding, we observed an increase in both MCP1 and S100A9 protein levels in the skin of the transgenic mice. Therefore, we believe that the increase in CD11b^+^Gr1^+^ cells in the skin of K14E7 mice could be promoted via MCP1 and S100A9; however, we have not excluded the possibility that other molecules are also involved.

## 5. Conclusion

Our data indicate that the expression of the HPV16 E7 oncoprotein in the skin affects the morphology and function of LCs and the proportion of CD11b^+^Gr1^+^ cells. These defects could lead to the local and systemic induction of an immunosuppressive environment.

## Supplementary Material

The morphology of MHC-II^+^ and CD205^+^ cells was also evaluated by IHC in hyperplastic skin from K14E7 mice. Similar to immunofluorescence results the LCs displayed a round shape and less number of dendrites.
