# Supplementary material for: The HPV16 E7 Oncoprotein Disrupts Dendritic Cell Function and Induces the Systemic Expansion of CD11b+Gr1+ Cells in a Transgenic Mouse Model
Source: Biomed Res Int. 2016 Jul 11;2016:8091353. doi: 10.1155/2016/8091353 (PMC4958469; doi:10.1155/2016/8091353)
Supplement: Supplementary file 1 — The morphology of MHC-II+ and CD205+ cells was also evaluated by IHC in hyperplastic skin from K14E7 mice. Similar to immunofluorescence results the LCs displayed a round shape and less number of dendrites. [file 8091353.f1.pdf]

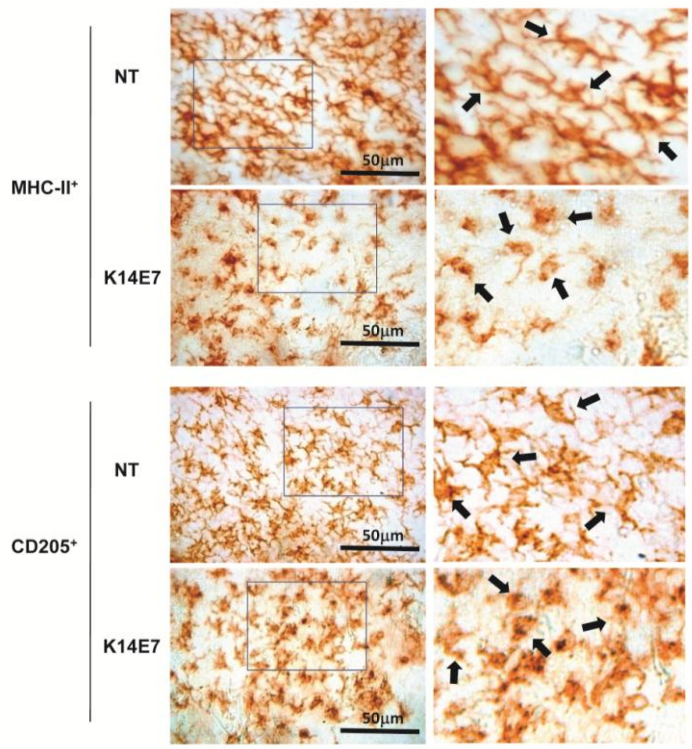

Supplementary figure. MHC-II$^+$ and CD205$^+$ cells in the epidermis of K14E7 transgenic mice. Representative images of MHC-II$^+$ cells with a dendritic appearance (black arrows) observed in epidermal sheets from 6-month-old NT (Non-Transgenic) mice. These cells exhibited a reduction in the number of visible dendrites (black arrows) in 6-month-old K14E7 mouse epidermal sheets. Similar morphological alterations were observed in CD205$^+$ cells in epidermal sheets derived from transgenic mice compared with NT mice. The results are representative of 4 mice per group.
